# B Cells Specific CpG Induces High IL-10 and IL-6 Expression In Vitro in Neuro-Behçet’s Disease

**DOI:** 10.3390/cells11081306

**Published:** 2022-04-12

**Authors:** Olfa Maghrebi, Meriam Belghith, Cyrine Jeridi, Amine Rachdi, Fatma Nabli Fatnassi, Zakaria Saied, Samir Belal, Samia Ben Sassi, Mohamed-Ridha Barbouche

**Affiliations:** 1 Department of Biology, Tunis El Manar University, Tunis 1068, Tunisia; olfamaghrebi3@gmail.com; 2 Laboratory of Transmission, Control and Immunobiology of Infections, Institut Pasteur de Tunis, Tunis 1002, Tunisia; medridha.barbouche@pasteur.tn; 3 Faculty of Medicine, Tunis El Manar University, Tunis 1007, Tunisia; samir_belal@ymail.com (S.B.); bensassisam@yahoo.fr (S.B.S.); 4 Neurology Department, Mongi Ben Hamida National Institute of Neurology, Tunis 1007, Tunisia; jeridicyrine@gmail.com (C.J.); dr.rachdi.amine@gmail.com (A.R.); fatmafatnassi@yahoo.fr (F.N.F.); zakariasaied@hotmail.com (Z.S.)

**Keywords:** Neuro-Behçet, multiple sclerosis, B cells, IL-10, IL-6

## Abstract

Remitting-RelapsingMultiple Sclerosis (RRMS) and Neuro-Behçet Disease (NBD) are two chronic neuroinflammatory disorders leading to neurological damage. Herein, we investigated in these patients the IL-10-producing cells during the early stages of these disorders. Cellular and molecular investigations were carried out on treatment naive patients suffering from RRMS and NBD recruited at the first episode of clinical relapse. Our findings demonstrate that CSF-B cells from NBD patients, but not RRMS, are the major source of intrathecal IL-10 as compared to T-CD4 cells. Moreover, we showed a lower expression of TGF-β and IL35, in the CSF cells of NBD patients as compared to the control group. Specific in vitro CpG stimulation of peripheral blood B cells from NBD patients resulted in a concomitant early mRNA expression of IL6 and IL10 but was limited to IL10 for RRMS patients. Furthermore, mRNA expression of IL-6 and IL-10 receptors was assessed and intriguingly IL6ST receptor subunit was significantly lower in NBD CSF, but not RRMS while IL10RB was increased in both. Deciphering the role of increased IL-10-producing B cells and IL10RB despite relapsing disease as well as the discordant expression of IL6 and IL6ST may pave the way for a better understanding of the pathophysiology of these neuro-inflammatory disorders.

## 1. Introduction

Neuroinflammatory diseases are generally triggered when the CNS is infiltrated by blood-borne leukocytes [1]. This inflammation is associated with the activation of CNS-resident immune cells, the release of proinflammatory cytokines, the production of reactive oxygen species and chemokines, and the infiltration of leukocytes through disturbed blood-brain barriers. All these events create a positive feedback loop in which continued recruitment and activation of leukocytes and glial cells can lead to sustained inflammation and long-term neuronal damage [2]. Multiple Sclerosis (MS) is defined as an immune-mediated neuroinflammatory neurodegenerative disorder of the central nervous system (CNS) characterized by mild pleocytosis and T cell infiltration in the cerebrospinal fluid (CSF) [3,4,5]. On the other hand, Neuro-Behçet’s disease (NBD), the neurological involvement of Behçet’s disease, is a multisystem auto-inflammatory disorder, associated with an inflammatory cascade and neutrophils infiltration in the CSF [6,7]. MS and NBD are multifactorial, complex, and heterogeneous with different clinical and pathological features. These disorders often afflict genetically predisposed individuals influenced by environmental factors and dysregulated immune activation. Viral Infectious agents have been reported to be associated with the etiologies of CNS diseases. Indeed, it was recently reported that the risk of MS increased 32-fold after infection with EBV but did not increase after infection with other viruses [8].

T helper 1 (Th1), and Th17 cells secreting IFN-γ and IL-17 pro-inflammatory cytokines, respectively, have been described as the major infiltrating lymphocytes within the CNS in neuroinflammatory diseases which is common to MS and NBD [9]. These cytokines orchestrate the inflammatory cascade and act on resident CNS cells to induce the production of IL-1β, IL-6, and TNF-α. Studies on MS and Behçet’s disease reported that Th1 and Th17 cells are involved in the persistence and the progression of the disease [10,11,12,13,14,15]. Compared to MS, NBD remains understudied in terms of immunopathogenesis, biologic markers, and potential treatment targets.

Indeed, the activation of CD4(+) T cells within the CNS leads to the recruitment of other cells, such as B cells, which could promote inflammation through the secretion of autoantibodies, pro-inflammatory cytokines, or via antigen-presentation. The role of B cells as a producer of proinflammatory cytokines and promoting the pathogenesis of chronic neuroinflammation has set the stage after the success of the antiCD20 therapy in patients suffering from multiple sclerosis [16]. B cells from MS patients were described to secrete proinflammatory cytokines [17]. However, a subset of B cells has been described to play a key role in regulating the immune responses to pathogens and autoantigens. This population of regulatory B cells (Bregs) secretes predominantly IL-10, TGF-β and IL-35. IL-35 is a heterodimeric cytokine composed of two subunits IL-12p35/L-12α and IL-27β chains Ebi3 encoded by IL12A and EBI3 (Epstein-Barr virus-induced gene 3), respectively. It was shown that IL-10-producing B cells are necessary for recovery in experimental autoimmune encephalomyelitis (EAE) [18]. The balance between pro and anti-inflammatory cytokines secreted by a cell could be assessed by the study of its receptors [19]. Since both IL-10 and IL-6 signaling is primarily transduced through STAT3, the study of their receptor emerged as a convenient way to appreciate the responsiveness of the cells to these cytokines [20]. To the best of our knowledge, no data are available on autoinflammatory diseases.

IL-10 was described as the major cytokine, which limits and prevents neurological damage and resolves acute inflammatory phases but intriguingly we found that it was increased in CSF of relapsing NBD. Most investigations were mainly focused on the production of IL-10 by innate resident cells, microglial cells, and astrocytes [21]. Regulatory high producing IL-10 B and T cells have been reported in other neuroinflammatory disorders, but not in CSF of NBD patients. Along with IL-10, IL-6 was described to be elevated in NBD patients and to contribute to the disease progression [22]. In our study, we aim to analyze the contribution of cells in IL-10 production in both blood and CSF of patients suffering NBD as compared to RRMS. In a second step, we examined the potential co-expressed cytokines with IL-10 and explored the IL-6 and IL-10 receptor expression.

## 2. Materials and Methods

### 2.1. Patients

All recruited patients were sampled during the first clinical episode of acute disease before any immunomodulatory treatment. All patients were enrolled from the Neurology Service of Mongi Ben Hamida National Institute (Tunis, Tunisia), during 2017–2020. Subjects were diagnosed based on clinical examination, magnetic resonance imaging (MRI), and CSF laboratory data (IgG index, oligoclonal IgG band).

For this study, we recruited 38 Neuro-Behçet’s patients; 27 patients were enrolled for the molecular exploration and 11 for the cellular studies and 50 patients suffering from Multiple Sclerosis divided into 27 included for molecular investigation and 22 for cellularstudies. The Neuro-Behçet group included in our study has a parenchymal involvement and has fulfilled the International Study Group Criteria for Behçet’s disease [23]. Patients with a non-parenchymal form of NBD were excluded from the study. Multiple sclerosis patients have remitting relapsing form (RRMS) and have fulfilled the revised McDonald Criteria [24] All MS patients met the following inclusion criteria: age > 18 years old; have the relapsing-remitting (RR) form of the disease; they underwent conventional MRI (3Tesla) with Gd-enhancement and lumbar puncture and showed positive oligoclonal bands restricted to CSF by isoelectric focusing. They were examined and scored according to Kurtzke’s Expanded Disability Status Scale (EDSS) to assess the clinical severity of the disease. MS patients presenting a primary progressive form were not included. The Control group consists of 24 subjects with non-inflammatory neurological diseases (NIND) with a persistent headache that requires a lumbar puncture. Suspicions for those controls of auto-immune or inflammatory neurological diseases are discorded after further analysis. Due to the limited CSF cell count, we were confronted to divide patients (RRMS and NBD) into two groups. For the first group of patients, we performed molecular exploration, and for the second group, we performed cytometric analysis by using the whole sample of CSF. Demographic, biological, and clinical characteristics of patients enrolled in this study are summarized in Table 1.

The ethical approach of this study was approved by the institutional ethical committee and written informed consent was obtained from all participants before the inclusion in the study (ethical committee code: IPT/PCI-LR11IPT02/22/2013).

### 2.2. Sample Collection and Processing

Blood and cerebrospinal fluid (CSF) of controls (NIND) and patients suffering from inflammatory disorders of the CNS were collected. Patients were sampled at the first time of clinical relapse. Up to 5 mL of peripheral blood was collected by venipuncture and up to 3 mL CSF was collected by lumbar puncture on ice. Peripheral blood mononuclear cells (PBMCs) were immediately isolated by Ficoll-Hypaque (Eurobio, Paris, France) density gradient centrifugation. CSF was centrifuged at 1650 rpm for 10 min at +4 °C to isolate the cells. PBMCs and CSF cells pellets were resuspended in RPMI 1640 medium supplemented with 10% heat-inactivated FCS, 1.5 mM L-glutamine, 100 U/mL penicillin and 100 µg/mL streptomycin (Gibco BRL–Life Technologies, Grand Island, NY, USA) for intracellular staining and functional study. For the group of patients intended for molecular investigations, purified PBMCs were stored in Trizol reagent (Sigma-Aldrich, Taufkirchen, Germany) and CSF cells were resuspended in an RLT buffer supplemented with 2% of beta mercapto-ethanol (QIAGEN, Venlo, The Netherlands). These samples were stored at −80 °C for ensuing RNA extraction.

### 2.3. Intracellular Il-10 Staining

Freshly isolated PBMCs and CSF cells were activated for six hours with PMA (50 ng/mL, Sigma-Aldrich), ionomycin (500 ng/mL, Sigma-Aldrich), and Golgiplug (BD Biosciences, Paris, France) before flow cytometric analysis. Cells were immunostained by surface antibody anti-CD19 (HIB19) and anti-CD4 (RPA-T4) conjugated respectively with Fluorescein isothiocyanate (FITC) and Allophycocyanin (APC) (BD Biosciences, Paris, France). Cells were fixed and permeabilized according to Cytofix/Cytoperm™ Kit protocol (BD Biosciences, Paris, France). Intracellular Phycoerythrin (PE) conjugated anti-IL-10 antibody (JES3-9D7) was added to cell suspension for 1 h in dark at +4 °C (BD Biosciences, Paris, France). Data were analyzed using the FlowJo software (version 7.6).

### 2.4. IL-10 and IL-6 Expression Kinetics

Freshly isolated PBMCs were adjusted to 1 × 10^6^ cells/mL and were seeded onto 24-well flat-bottom plates (Corning, New York, NY, USA) in presence of 3 μg/mL of CpG ODN 2006 (CarthaGenomics, Tunis, Tunisia) or left unstimulated at 37 °C with 5% CO_2_. Cells were harvested at 1 h, 3 h, 6 h and 24 h after stimulation and stored in RLT supplemented with 2% of beta mercapto-ethanol at −80 °C for subsequent RNA extraction. Quantitative real-time PCR analysis was carried out to assess the IL-10 and IL-6 induction using the appropriate primers upon the stimulation with CpG ODN 2006.

### 2.5. RNA Extraction and Quantitative Real Time PCR

RNAeasy mini kit (QIAGEN, Venlo, The Netherlands) was used to extract RNA from PBMCs and CSF cells. The extracted RNA was controlled in terms of quality and quantity using agarose gel electrophoresis and NanoDrop respectively. Reverse transcription was performed on DNase I treated RNA utilizing High-capacity cDNA reverse transcription kit (Applied Biosystems by Thermo Fisher, Vilnius, Lithuania) following the manufacturer’s recommendations. Quantitative real-time PCR was carried out using SyberGreen technology on the Applied Biosystems ABI PRIZM 7500 Real-Time PCR System following the protocol described by Belghith et al. 2018 [25]. In the present study, we quantified genes encoding for regulatory cytokines TGF-β and IL-35 including the two subunits IL-12p35 and IL-27 β namely TGFB, IL12A, and EBI3. Furthermore, we quantified the genes encoding for IL-6 (IL6) and its receptor composed by gp130 (IL6ST) and IL-6rɑ (IL6R), and IL-10 (IL10) and the two subunits IL-10Rɑ (IL10RA) and IL-10Rβ (IL10RB). Glyceraldehyde-3-phosphate dehydrogenase (GAPDH) was used as an endogenous reference to perform Relative quantification of mRNA run in duplicate in three independent experiments. The sequences of the employed primers are consigned in Supplementary Table S1. Randomly, the obtained PCR products were supplementary checked on an agarose gel.

### 2.6. Statistical Analysis

GraphPad Prism version 8.0.0 for Windows, GraphPad Software, San Diego, CA, USA was used for all the graphs, calculations, and statistical analyses. The Mann–Whitney U test and Wilcoxon signed-rank test were used to compare between two groups and Kruskal-Wallis test for three or more groups with nonparametric data. Categorical variables describing characteristics of patients and controls were compared with contingency tables. Values of *p* ≤ 0.05 were considered significant.

## 3. Results

### 3.1. CD19 B Cells Are a Major Contributor to IL-10 Production in the CSF of NBD

To better characterize the source of highly expressed IL-10 reported in the CSF of parenchymal-NBD patients as compared to RRMS patients [14]. We herein confirmed by flow cytometry high intracytoplasmic IL-10 expression in blood and CSF cells from NBD patients. In addition, we sought to investigate the cellular origin of IL-10 by studying IL-10-producing B and CD4+T cells subsets at the first episode of the clinical symptoms of NBD as compared to RRMS and NIND.

**Figure 1:**
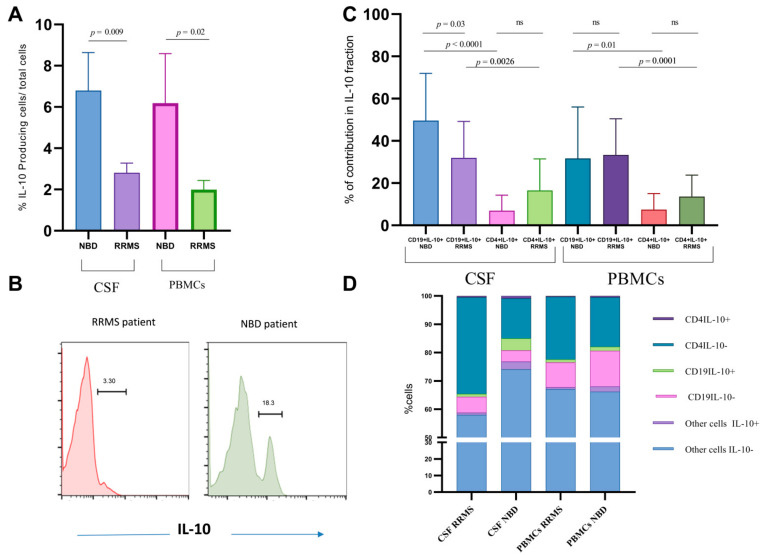
Flow cytometry analysis of IL-10-producing cells in CSF and total PBMCs. (**A**) Comparative histogram of IL-10 frequencies within the CSF and total PBMCs from RRMS (*n* = 22) and NBD (*n* = 11) patients. (**B**) A Representative intracellular expression of IL-10 in the total CSF of RRMS and NBD patients. (**C**) Histogram representation of comparative contribution of CD19 and CD4 cell subsets in CSF and PBMCs of NBD and RRMS patients. The results are mean ± SEM with. Boxplots represent the percentage of IL-10-producing CD19 and CD4 cells in the CSF and PBMCs of neuro-immunological disorders RRMS (*n* = 22) and NBD (*n* = 11). Statistical significance between two groups was assessed using the Wilcoxon–Mann–Whitney test. (**D**) Bar charts plots of the percentages and frequencies of IL-10 positive cells in the whole CSF and blood. Representation ofCD4 IL-10+ cells, CD4 IL-10- cells, CD19 IL-10+ cells, CD19 IL-10- cells and the otherundetermined cells secreting or not IL-10 of NBD and RRMS patients. ns = non significant.

Total CSF cells of NBD patients in comparison with CSF cells of RRMS showed that IL-10 levels were significantly higher in NBD than in RRMS patients (*p* = 0.0098) (Figure 1A). A representative histogram of IL-10 intracytoplasmic expression in NBD versus RRMS is shown in Figure 1B. This pattern was also observed in the PBMCs of the two groups of patients (*p* = 0.0194) (Figure 1A). In contrast, the gating on IL-10+ cells in the PBMCs showed no difference in IL-10 production by CD4 and B (Figure 1C), suggesting that in blood other cells significantly contribute to IL-10 production. In the CSF, higher CD19+/IL-10+ B cells percentage was observed in NBD as compared to RRMS (*p* = 0.0382) (Figure 1C). The distribution of IL-10+/CD19+ B cells is significantly higher than that of IL-10+/CD4+ T cells in CSF of NBD patients as compared to RRMS patients (*p* < 0.0001), reflecting a stronger contribution of CSF B cells to IL-10 production in the NBD group.

The representation in Figure 1D of respective B, T and other cells contribution to IL-10 production highlights the predominant presence of IL-10-producing B cells in the CSF of NBD. Interestingly, we noted in the CSF and PBMCs of the NBD patients the presence of an IL-10-producing population that was neither CD19 nor CD4. This fraction of non B non T IL-10+ cells represents 2.7% in CSF and 1.78% in PBMCs of NBD and is higher than that observed in RRMS (*p* = 0.003) (Figure 1D).

### 3.2. IL-10 Expression Is Not Associated with the Regulatory Markers TGF-β and IL-35

**Figure 2:**
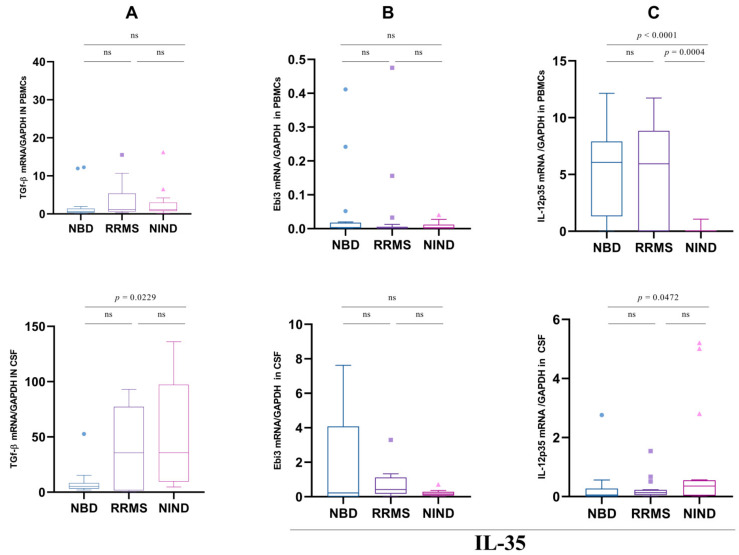
Boxplots representation of CSF and PBMCs regulatory cytokines expression in patients and NIND. (**A**) Representative boxplots of TGF-β expression in the PBMCs and CSF cells of NBD (*n* = 25), RRMS (*n* = 22) and NIND (*n* = 23) (**B**) IL12-p35 expression in PBMCs and CSF cells per group. (**C**) Representative boxplots of Ebi3 expression in PBMCs and CSF cells of NBD, RRMS and NIND. Statistical significance between two groups was assessed using the Wilcoxon–Mann–Whitney test. ns = non significant.

Generally, IL-10 secretion is the hallmark of a specific B cell population called Breg cells. Along with IL-10, IL-35 and TGF-β are effective cytokines secreted by Breg which contribute to fine-tuning immune responses and preventing tissue damage. We then investigated the mRNA expression levels of the genes encoding for TGF-β and the two subunits of IL-35: Ebi3 and IL-12p35. These analyses were performed on PBMCs and CSF of the two groups of patients and the control group addressed as NIND (non-inflammatory neuro-immunological disorders). We noticed, in PBMCs, no significant differences in the TGFB mRNA expression between the three studied groups (Figure 2A). Conversely, in the CSF, we observed a significant decrease of mRNA TGFB expression in the NBD patients as compared to the NIND control group (*p* = 0.0229) (Figure 2A). When analyzing the expression profile of the two subunits of the IL-35 cytokine in the samples of the three studied groups, we noticed no significant differences in the EBI3 expression in the PBMCs (Figure 2B), among the three groups. We noticed in the CSF that EBI3 mRNA was slightly increased in NBD compared to RRMS. However, we observed a significant decrease in IL-12A mRNA expression in the CSF of the NBD group as compared to the NIND group (*p* = 0.0472). Interestingly, in PBMCs, IL-12A was significantly increased in RRMS and NBD patients as compared to controls (*p* = 0.0004; *p* < 0.0001) (Figure 2C).

### 3.3. Kinetics of IL10 and IL6 Expression

In the CSF of NBD, we showed increased IL-10-producing B cells, without elevated TGF-β nor IL-35 expressions. B cells may also secrete pro-inflammatory cytokines in response to various stimuli. TLR9 signaling mediates the activation of B cells by increasing secretion of pro-inflammatory (IL-6) and immune regulatory (IL-10) cytokines [26].

**Figure 3:**
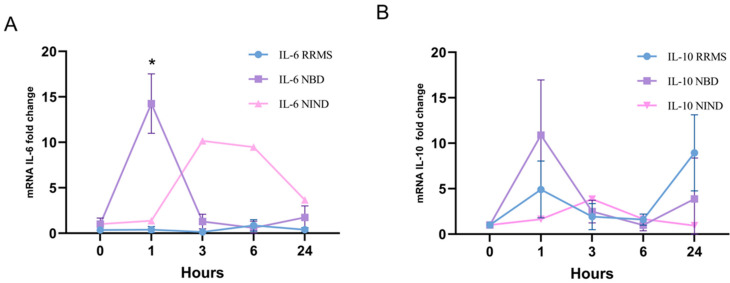
Dynamics of IL10 and IL6 expressions in RRMS and NBD patients. (**A**) IL6 induction in PBMCs of RRMS, NBD and NIND at 1, 3, 6 and 24 h following TLR9 ligation by CpG ODN2006. The results are mean ± SEM with * *p* ≤ 0.05 a minimum of three independent experiments. (**B**) IL10 induction by CpG in PBMCs of RRMS, NBD and NIND at 1, 3, 6 and 24 h following TLR9 ligation by CpG ODN2006. Statistical significance was assessed using nonparametric multiple *t*-test and Kruskal–Wallis tests.

Upon stimulation by CpG we observed a peak expression occurring after 1 h for both IL6 and IL10 from PBMCs of NBD patients. For the NIND group, the highest induction of IL10 and IL6 expressions was reported after 3 h of stimulation. Interestingly, we observed a lower induction of Il-6 expression at 1 h of PBMCs ‘CpG stimulation in RRMS as compared to PBMCs from NBD patients (*p* = 0.013) (Figure 3A). However, for IL10 induction, no significant difference was noted between the two groups of neuroinflammatory disorders (Figure 3B). Taken together, these results showed a concomitant early expression of IL10 and IL6 in patients suffering from NBD in contrast to RRMS patients.

### 3.4. Differential Expression of IL-10 and IL-6 Receptors

By further exploring the signaling pathways of IL-6 and IL-10, we performed in the CSF, the mRNA relative quantification of the subunits of IL10 and IL6 receptors. Genes encoding forIL10Rα, IL10Rβ, IL6Rα, and gp130 were assessed by quantitative real-time PCR in the PBMCs and CSF of the studied groups (RRMS, NBD and NIND).

**Figure 4:**
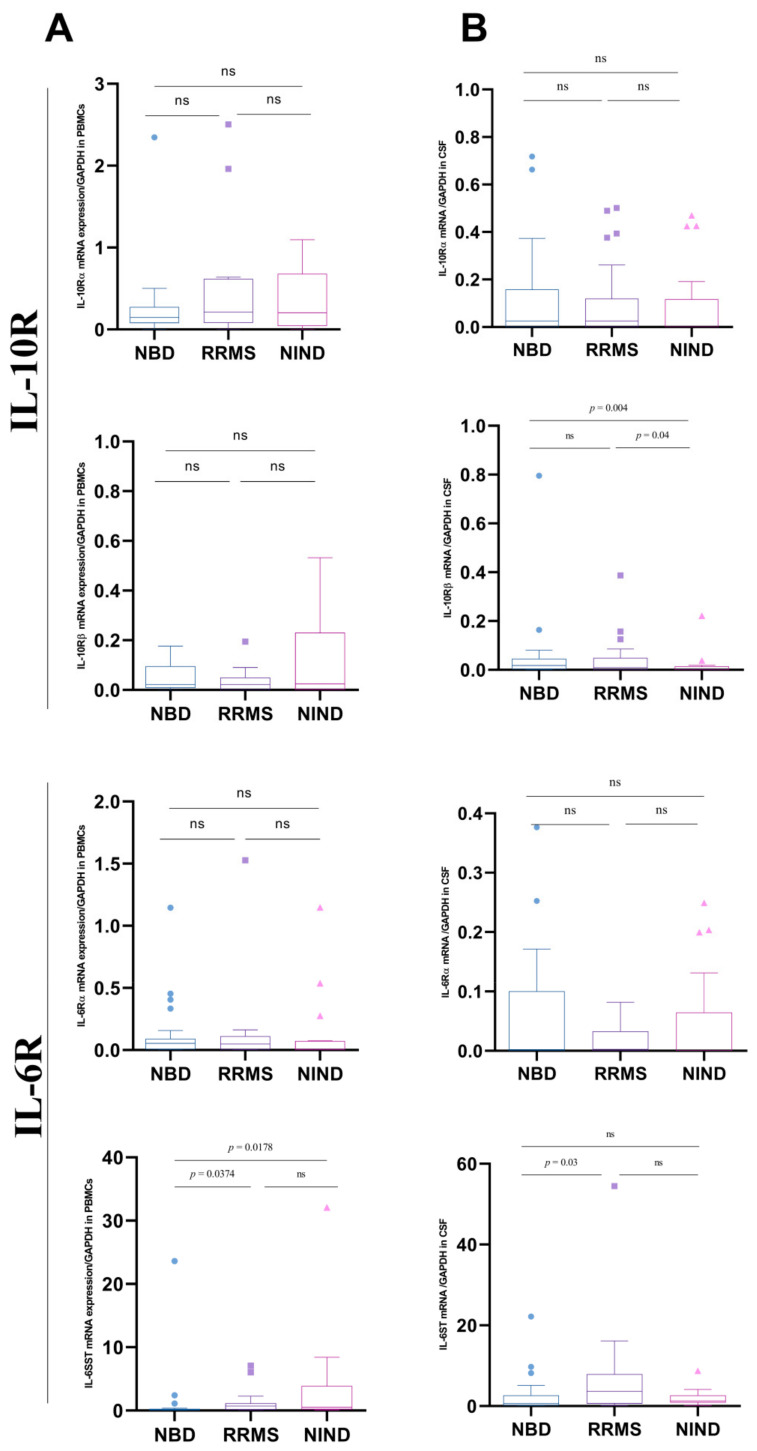
Boxplots representation of subunits of IL-10 and IL-6 receptors expression in CSF and PBMCs cells of patients and controls. (**A**) Blood expression of IL10RA, IL10RB, IL6R, and IL6ST in NBD (*n* = 21), RRMS (*n* = 23) and NIND (*n* = 19). (**B**) CSF expression of IL10RA, IL10RB, IL6R, and IL6ST in NBD, RRMS and NIND. Statistical significance between the two groups was assessed using the Wilcoxon–Mann–Whitney test; ns = non significant.

In PBMCs, we did not detect any significant difference in the expression levels of the two subunits of the IL-10 receptor, between the three studied groups. Though studying the two subunits of the IL6 receptor in the same compartment, we noted that the IL6RA was equally expressed in the three groups while the IL6ST was decreased in the NBD group as compared to the RRMS patients (*p* = 0.0374) and the NIND group (*p* = 0.0178) (Figure 4A).

We performed in the CSF, the mRNA relative quantification of the subunits of IL-10 and IL-6 receptors. For the IL10, we found an increase in IL10RB in the two groups of patients as compared to controls (NBD versus NIND *p* = 0.0045); (RRMS versus NIND *p* = 0.0403). Concerning the IL6 receptor subunits, IL6ST was found to be more expressed in the group of RRMS patients as compared to NBD (*p* = 0.0296) (Figure 4B).

## 4. Discussion

The cellular source of IL-10 in the cerebrospinal fluid of neuroimmunological disorders is poorly studied. Here, we investigated the IL-10-producing cells in the blood and CSF of RRMS and NBD patients, enrolled at the first episode of the disease before any treatment. We first analyzed cellular secretion of IL10 and showed in NBD CSF patients, a higher proportion of IL10 producing cells as compared to RRMS patients. Moreover, our data argue in favor of B cells as being the predominant secretors of IL10 in NBD CSF while both T CD4 and B cells secrete IL-10 in RRMS CSF. Elevated levels of IL-10 have been reported in chronic inflammatory diseases such as systemic lupus erythematosus (SLE), rheumatoid arthritis (RA), systemic sclerosis (SSc), and adult-onset Still’s a disease (AOSD) [27]. In the model of BD, elevated IL-10 levels have been reported in patients’ lesions [28].

The central nervous system has been proposed as a fostering environment for B cells in a variety of neurological disorders, including multiple sclerosis [29,30]. The trafficking of B cells into the CNS is guided by several chemokines, which control migration across endothelial barriers. Previous studies on MS and NB patients showed increased levels of CXCL9/CXCL10 chemokines associated with B cells migration [31,32,33]. Furthermore, CXCL13, responsible for follicle formation and differentiation of B cells [33,34,35,36], and BAFF(B-cell activating factor), promoter of long-term survival of B cells, were shown to be up-regulated in both RRMS and NBD [37,38,39,40]. Other studies indicated that the majority of RRMS patients have elevated CSF B cell levels as compared to progressive MS and other neurological diseases. The follow-up of these patents showed that the frequency of B cells in the CSF of MS patients is inversely correlated with the disease severity [41,42,43]. The most frequent B cell subpopulation in MS CSF was CD19+CD138− mature B cells followed by CD19+CD138+ plasmablasts [41]. On the other hand, in Behçet’s disease, B cells play an active role in the inflammatory process asCD20+ B cells were detected in a ruptured pulmonary artery aneurysm [44]. The up-regulated mRNA expression of BAFF was described in three cases of Neuro-Behçet skin biopsies. Moreover, previous results showed an intrathecal increased production of BAFF in NBD CSF patients [37]. The increased proportion of IL-10-producing B cells in the CSF of NBD described in our study is in accordance with the data reported by Yang et al. who showed that BAFF may lead to an expansion of IL-10-producing B cell subpopulation by several potential mechanisms, including differentiation of regulatory B cells or BAFF-induced proliferation or protracted survival of this subpopulation [45].

B regulatory cells with anti-inflammatory capacity have been shown to inhibit the expansion of inflammatory T cells through the production of IL10 and IL-35 and TGF-β. We demonstrated lower levels of TGF-β in CSF of NBD patients as compared to controls and no difference in the blood compartment of the three studied groups. The lower expression of TGF-β observed in NBD CSF is consistent with a previous report that showed decreased levels of the secreted TGF-β cytokine [46]. TGF-β producing B regs were shown to induce apoptosis of CD4+T cells and cell anergy in CD8 T effector cells [47]. This growth factor plays an essential role in tolerance by inducing T cells into Foxp3 Tregs. The expression of TGF-β observed in the CSF and blood compartments of RRMS patients is consistent with a previous report showing a correlation between enhanced levels of this growth factor and disease progression [48]. In our study, the patients were enrolled at the first episode of the disease and no CSF sample, other than the first one, was accessible to allow follow-up of this parameter.

The blood IL-10-producing B cells of RRMS patients herein observed as not significantly different from NBD (1.47% vs. 1.04%) were already described in MS patients not receiving rituximab [49]. Indeed, several studies have documented that naïve B cells are the predominant producers of IL-10 [50], and others showed that CD27+ memory B cells represent the main producers of this cytokine [51].To our best knowledge, secreting IL10 B cells during NBD has not been previously investigated.

The rationale behind the study of the expression of the two subunits of IL-35 (IL-12p35 and Ebi3) in the cerebrospinal fluid of RRMS and NBD patients, was the assessment of their effective co-expression since these subunits are shared by other cytokines. Our data indicate a significant decrease of IL-12p35 in the CSF of NBD compared to controls with no difference in the expression of the Ebi3 subunit. No correlation between the two subunits was observed. In contrast, we noted a significant increase of IL-12p35 in the blood of both NBD and RRMS as compared to the control group. The increased mRNA expression of IL-12p35 is in agreement with data already reported in the RRMS [52] and Behçet patients [53], suggesting a potential concomitant increase of IL12 cytokine.

Taken together, our data obtained in the CSF of NBD patients showed the existence of a population of B cells producing IL-10 with decreased expression of TGF-β. We could speculate that those IL-10-producing B cells may secrete other proinflammatory cytokines (i.e., IL-6, GM-CSF, TNF-α). Indeed, Lighaam et al. discussed that IL-10-producing B cells often co-express pro-inflammatory cytokines like IL-6 and TNF-α [26]. In this study, we focused on IL6 induction in B cells. IL-6 in the CNS was previously described as a biomarker that correlates with the NB disease activity [54]. In vitro stimulated CpG from NBD showed a concomitant higher early expression of IL-10 and IL-6, however, CpG from RRMS patients showed IL-10 secretion only by B cells with lower levels as compared to NBD. When cultured in vitro, B cells from MS patients exhibited a striking defect in their ability to secrete IL-10 cytokine after prolonged stimulation [55]. Furthermore, the lower induction of IL-6 in RRMS may be partially explained by the reduced expression level of the CpG target, the TLR9, in memory B cells known to be increased in MS patients. Indeed, inducing IL-6 production by these memory B cells in RRMS patients requires a combination of a high-dose CD40L and additional stimulatory molecules [56].

When assessing the expression of the receptors of IL-10, we noted an increase of IL10RB in CSF of NBD and RRMS as compared to NIND. We could speculate that this increase is associated with the presence of cytokines sharing the same receptor subunit e.g., IL-26, which was described to be increased in the CSF of NBD and untreated MS [57,58]. Interestingly, we also report an over-expression of IL6ST (gp130) in RRMS patients compared to NBD in the blood and the CSF. It was previously described that gp130 expression mediates CNS-infiltration and causatively induces EAE by particularly promoting the early development of pathogenic TH17 cells [59]. The differential expression of gp130 between the two disorders could explain the increased intrathecal expression of Ig found in RRMS and the presence of OCB while gp130 signaling via STAT3 in B cells promotes long-lived plasma cells and Ig production [60,61,62].

Our data demonstrate that intrathecally produced IL-10 is mostly attributed to B cells than to TCD4. Given the ability of cytokine-expressing B cells to modulate immune responses in an antibody-independent manner, elucidating cytokine phenotype of those cells and the underlying pathways are of considerable interest not only in understanding the pathogenesis of neuroinflammatory disorders but also for the identification of novel therapeutic strategies.

Furthermore, after the striking success of rituximab (anti-CD20) in various inflammatory neurological disorders, the B cell role in the pathophysiology of these two disorders (MS and NBD) gained much more attention. This treatment has already been proven to have beneficial effects in patients with inflammatory demyelinating disorders including MS [52]. Anti-CD20 significantly reduced the number of gadolinium-enhancing lesions, the number of relapses in RRMS patients [16] and led to the expansion of a rare regulatory B cell population [63]. Interestingly, rituximab was also reported as efficient in severe ocular manifestations of Behçet’s disease [64] and two cases of neuro-Behçetpatients presenting pseudotumoral lesions and one with relapsing NBD [65,66]. No studies exploring the underlying causes of rituximab efficiency in these rare cases of NBD were performed. Mechanisms of action on B cells might be different in these two diseases. Our Results plead in favor of B Cells being a promising therapeutic target in Neuro-Behçet even in the early stages. Nevertheless, we admit that this study is subject to limitations. Indeed, due to sampling constraints and the limited amount of cells in CSF several phenotypical and functional characteristics of the B cell were not extensively studied. We also acknowledge that a higher number of patients would increase the statistical power of this study.

## 5. Conclusions

In conclusion, the most striking finding in this study is the presence of IL-10 producing cells in the CSF of NBD and RRMS patients. We found a major contribution from B cells to this secretion of IL-10 in the CSF of NBD patients but not RRMS. This predominance of IL-10-producing B cells in NBD CSF did not correlate with either TGF-β nor IL-35 suppressive cytokines expression. Following CpG activation of peripheral B cells, we observed a concomitant expression of both IL10 and IL6 in NBD but not in RRMS B cells, while the latter express mainly IL10. Finally, the discrepancies we report in the expression of the IL6ST receptor subunit between both diseases may be relevant to the underlying inflammatory process. Taken together, these novel findings may pave the way to further studies aimed to better understand the role of these in situ IL-10-producing B cells in the balance between immunosuppressive and inflammatory processes governing the immunopathology of these two diseases.

## Figures and Tables

**Table 1:** Demographic, biological and clinical characteristics of patients.

	Group of Patients for Molecular Exploration				Group of Patients for Cellular Exploration		
Disease	NBD	MS	NIND	*p*	NBD	MS	*p*
Number of patients	27	28	24		11	22	
Sex ratio (F/M)	(11/16)	(23/5)	(21/3)	0.0003	(5/6)	(12/10)	0.72
Mean age	42.65	39.4	42.52	0.66	29.42	34	0.28
(sd)	(±11.47)	(±10.58)	(±20.1)		(±9.03)	(±12.07)	
IgG Index	0.52	0.931	0.42	<0.0001	0.53	1.08	<0.0001
(sd)	(±0.36)	(±0.49)	(±0.05)		(±0.04)	(±0.05)	
CSF/serum albumin ratio (10^−3^)	5.27	5.15	4.69	0.51	3.56	4.61	0.17
	(±1.488)	(±1.788)	(±2.059)		(±1.468)	(±1.335)	
Cell count	0.96 × 10^6^	0.34 × 10^6^	0.18 × 10^6^	<0.0001	0.99 × 10^6^	0.32 × 10^6^	<0.0001
EDSS		22			-	2	
Form of the disease	Parenchymal	Relapsing	-		Parenchymal	Relapsing	
		-remitting				-remitting	
Patients in relapse	all	all	-		all	all	
Patients under therapy	none	none	-		none	none	

NBD = neuro-Behçet disease; RRMS = relapsing-remitting multiple sclerosis; NIND = non-inflammatory neurological disease; F = female; M = male; EDSS = the Expanded Disability Status Scale; CSF, cerebrospinal fluid. Categorical variables were calculated via Chi-square, Kruskal–Wallis test was used to compare 3 groups, and Independent Mann-Whitney was used to compare 2 groups for continuous variables. Statistical significance was defined at *p* < 0.05.

## Data Availability

The data presented in this study are available on request from the corresponding author.

## Supplementary Materials

The following supporting information can be downloaded at: https://www.mdpi.com/article/10.3390/cells11081306/s1, Table S1: primers used in quantitative real-time PCR.
