# Supplementary material for: B Cells Specific CpG Induces High IL-10 and IL-6 Expression In Vitro in Neuro-Behçet’s Disease
Source: Cells. 2022 Apr 12;11(8):1306. doi: 10.3390/cells11081306 (PMC9025002; doi:10.3390/cells11081306)
Supplement: Supplementary file 1 [file cells-11-01306-s001.zip › cells-1564485-supplementary.pdf]

Supplementary Table S1: primers used in quantitative real time PCR

Gene	forward	Reverse
TGF-β	5'-GCCCTGGACACCAACTATTG-3	5'-CTGGTCCAGGCTCCAAAT-3'
Il-12p35	*5'-CCACTCCAGACCCAGGAATGT-3'*	5'-CCTCCACTGTGCTGGTTTTATCT-3'
Ebi3	5'-TCATTGCCACGTACAGGCTC-3'	5'-GGGTCGGGCTTGATGATGTG-3'
Il-6	5'-ATGAACTCCTTCTCCACAAGCGC-3'	5'-GAAGAGCCCTCAGGCTGGACTG-3'
Il-6st	5'-CACCCTGTATCACAGACTGGCA-3'	5'-TTCAGGGCTTCCTGGTCCATCA-3'
Il-6rα	5'-GACAATGCCACTGTTCACTG-3'	5'-GCTAACTGGCAGGAGAACTT-3'
Il-10	5'-CGAGATGCCTTCAGCAGAGT-3'	5'-CCCTTAAAGTCCTCCAGCAA-3'
Il-10rα	5'-GCTCCTGAGGTATGGAATAGAGTCC-3'	5'-TATGTGTCATTTGCGGGGGC-3'
Il-10rβ	5'-TGGATGACACCATTATTGGACCC-3'	5'-TTTGCTCACAGACAGGCTCACT-3'
GAPDH	5'-CCACATCGCTCAGACACCAT-3	5'GGCAACAATATCCACTTTACCAGAGT-3'
